# Aberrant Breast in a Rare Site: A Case Report

**DOI:** 10.1155/2012/943102

**Published:** 2012-06-26

**Authors:** Levent Yeniay, Kilongo Mulailwa, Elmir Asgerov, Cüneyt Hoşcoşkun, Osman Zekioğlu

**Affiliations:** ^1^Department of General Surgery, Ege University School of Medicine, Izmir, Turkey; ^2^Department of Pathology, Ege University School of Medicine, Izmir, Turkey

## Abstract

Aberrant breast tissue is an anomaly in the embryogenesis of the breast that is found along the mammary ridge or out of that line. We report a case of a 71-year-old female patient with an abdominal aberrant breast tissue found incidentally in a piece of mesenteric biopsy. The histological features were consistent with breast tissue.

## 1. Introduction

The concept of polymastia and polythelia suggests the presence of abnormally located breast tissue with or without the presence of the areola or nipple. It is generally located along the mammary ridge, running from the axilla to the groin; uncommonly when it is located out of that line, it is then referred to as “mammae erraticae.” Up to the best of our knowledge, only one case of aberrant breast tissue occurring in internal organs has been documented. Hereby, we report a case of aberrant breast tissue found incidentally on a piece of mesenteric biopsy in a female patient operated for an abdominal mass, owing to a recurrent endometrial malignant tumor.

## 2. Case Report

A 71-year-old woman, who underwent a total hysterectomy and oophorectomy 8 years ago for endometrial cancer, was admitted for recurrent abdominal mass detected during a routine control thoracoabdominal computerized tomography (CT) scan. CT scan revealed a 5 × 6 cm sized cystic mass located in the left para-aortic area, at the level of the renal artery orifice of the abdominal aorta. No other metastasis had been demonstrated. In the blood chemistry, only mild elevation of CA 125 was present. The rest was unremarkable.

**Figure 1:**
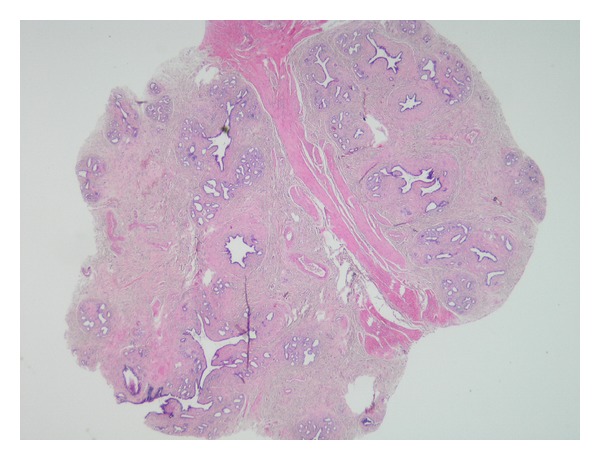
Lobular units with ductal structures in a fibrovascular tissue (x20).

**Figure 2:**
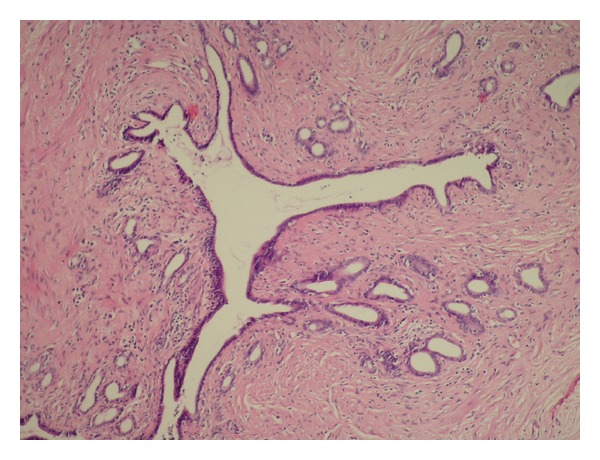
Ductal epithelial and acinar group in a fribrovascular stroma (x100).

**Figure 3:**
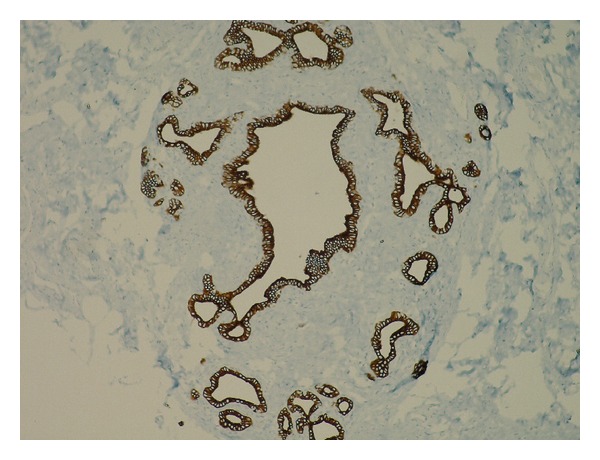
Result for immunohistochemical stain: diffuse epithelial stain for MFGP 1.

 A 6 × 6 × 5 cm sized tumoral mass, located between the abdominal aorta and the left renal artery, was revealed at the laparotomy. In addition, about 10 cm proximal to the ileocecal valve, a nodular soft and freely mobile mass of 3 cm diameter was observed on mesentery. A surgical resection of the para-aortic mass and an excisional biopsy of the mesenteric mass were performed. The histological evaluation of the para-aortic cystic mass revealed the metastasis of the primary endometrial serous papillary carcinoma. The histological features of the mesenteric mass were consistent with breast tissue, including lobular and ductal structures in a fibrovascular tissue (Figures 1 and 2). Immunohistochemistry with milk fat globulin protein 1 antibodies (MFGP 1) were positive for epithelial and acinar cells, consistent with mammary epithelial differentiation (Figure 3).

## 3. Discussion

The term ectopic breast tissue, which represents supernumerary breast, with or without the presence of a nipple or an areola [1], is an anomaly in the embryogenesis of the breast, which is most frequently encountered in females. The incidence is reported to be 1% up to 6% [2–5], with a sporadic mode of transmission, but unusually hereditary transmission in familial cases is possible [6], with variable penetrance [1, 6].

Such ectopic tissue usually is found within the milk ridge extending from the axilla to the groin [2–4]; less commonly, it appears in locations outside of the mammary ridge: the face, the neck, the chest, middle back buttock, anus, vulva, flank, hip, thigh, foot, shoulder. Upper extremities also have been described as locations of aberrant breast tissue [1, 2]. Only one case of heterotropic breast tissue developing in internal organ has been reported in the literature [3]. To the best of our knowledge, this is the first reported aberrant breast tissue in the abdomen.

Symptoms and signs of the ectopic breast tissue appear during puberty or pregnancy, which are correlated with hormonal effect on the breast tissue. The ectopic tissue is then found as a mass with intermittent volume changes between two menstruations or during the pregnancy. In our case, the deep location of the mass might have hidden the clinical features. Furthermore, no intermittent abdominal pain was present in our case. When the location of the ectopic breast tissue is out of the milk ridge, the diagnosis is rarely made on clinical ground [7].

Ectopic breast tissue can undergo the same pathologic changes as normal breast, including carcinomas [2]. Histological features found in the reported case can be considered as fibrovascular change of the breast tissue.

In conclusion, ectopic breast tissue is not an uncommon finding, but the variability of its location often explains the delay in its diagnosis. The treatment remains the surgical resection, for cosmetic or clinical purposes.

## References

[1] Yaghoobi R, Bagherani N, Mohammadpour F (2009). Bilateral aberrant axillary breast tissue. *Indian Journal of Dermatology, Venereology and Leprology*.

[2] Grossl NA (2000). Supernumerary breast tissue: historical perspectives and clinical features. *Southern Medical Journal*.

[3] Sasaki K, Parwani AV, Demetris AJ, Sasatomi E (2010). Heterotopic breast epithelial inclusion of the heart: report of a case. *American Journal of Surgical Pathology*.

[4] Chan NG, Penswick JL, Labelle E, Driman DK (2007). Ectopic breast tissue presenting as an anal polyp. *Canadian Journal of Surgery*.

[5] Conde DM, Kashimoto E, Torresan RZ, Alvarenga M (2006). Pseudomamma on the foot: an unusual presentation of supernumerary breast tissue. *Dermatology Online Journal*.

[6] Toumbis-Ioannou E, Cohen PR (1994). Familial polythelia. *Journal of the American Academy of Dermatology*.

[7] Hanief MR, Yongue G, Leff D (2011). painful perineal lump: an unusual case of ectopic breast tissue. *Annals of The Royal College of Surgeons of England*.

